# Preliminary Study of Structural Changes of Glucose-6-Phosphate Dehydrogenase Deficiency Variants

**DOI:** 10.37796/2211-8039.1355

**Published:** 2022-09-01

**Authors:** Naveen E. Louis, Muaawia A. Hamza, Puteri NSD Engku Baharuddin Baharuddin, Shamini Chandran, Nurriza A. Latif, Mona A. Alonazi, Khairul B.A. Halim, Arjumand Warsy, Syazwani I. Amran

**Affiliations:** a Department of Biosciences, Faculty of Science, Universiti Teknologi Malaysia, Johor Bahru, Johor, Malaysia; b Faculty of Medicine, King Fahad Medical City, Riyadh, Kingdom of Saudi Arabia; c Research Center, King Fahad Medical City, Riyadh, Kingdom of Saudi Arabia; d Department of Biochemistry, Science College, King Saud University, Riyadh, Kingdom of Saudi Arabia; e Department of Biotechnology, Kulliyyah of Science, International Islamic University Malaysia, 25200, Bandar Indera Mahkota Kuantan, Pahang, Malaysia; f Central Laboratory, Center Female Center for Scientific and Medical Studies, King Saud University, Riyadh, Kingdom of Saudi Arabia

**Keywords:** G6PD deficiency, Molecular dynamics simulation, Protein-ligand complex

## Abstract

Glucose-6-phosphate dehydrogenase (G6PD) deficiency is the most common enzyme deficiency disorder affecting over 400 million individuals worldwide. G6PD protects red blood cells (RBC) from the harmful effects of oxidative substances. There are more than 400 G6PD mutations, of which 186 variants have shown to be linked to G6PD deficiency by decreasing the activity or stability of the enzyme. Different variants manifest different clinical phenotypes which complicate comprehending the mechanism of the disease. In order to carry out computational approaches to elucidate the structural changes of different G6PD variants that are common to the Asian population, a complete G6PD monomer-ligand complex was constructed using AutoDock 4.2, and the molecular dynamics simulation package GROMACS 4.6.7 was used to study the protein dynamics. The G410D and V291M variants were chosen to represent classes I and II respectively and were created by *in silico* site-directed mutagenesis. Results from the Root mean square deviation (RMSD), Root mean square fluctuation (RMSF) and Radius of gyration (Rg) analyses provided insights on the structure – function relationship for the variants. G410D indicated impaired dimerization and structural NADP binding while the impaired catalytic activity for V291M was indicated by a conformational change at its mutation site.

## 1. Introduction

Glucose-6-phosphate dehydrogenase (G6PD) deficiency is the most common enzyme deficiency disorder affecting more than 400 million individuals worldwide [1]. With regards to global incidence of the enzymopathy, approximately 5–20% of cases are found in Asia [2,3]. G6PD is the key regulatory enzyme in the pentose phosphate pathway [4], responsible for the production of antioxidative component nicotinamide adenine dinucleotide phosphate (NADPH) which protects red blood cells (RBC) from the harmful effects of free radicals [5]. In the event of acquiring deleterious mutations which affect the topical structure of the protein, it would affect normal G6PD enzyme levels leading to a deficiency in the enzyme, and thus leading to RBC hemolysis under oxidative stress [6,7].

There have been reports of more than 400 G6PD variants, of which approximately 50% of variants lead to G6PD deficiency characterized by reduced enzyme activity and structural integrity of the protein structure [8]. G6PD variants resulting from different mutations lead to different clinical symptoms [9,10]. Furthermore, depending on the deleterious effects of a variant, they have been grouped into five classes (I, II, III, IV and V), classes I, II and III represent the most damaging variants, whereas classes IV and V are less harmful [9,11].

There are many mutations distributed throughout the protein structure [12], however the effects of these mutations on enzyme structure and function remain unclear. Less than 10% of known G6PD variants have been analyzed in depth by correlating their clinical manifestation with their respective mutations [13]. Hence, this study sought to investigate the structural–functional relationship of G6PD variants originating from Asia [14,15], using structural analysis and molecular dynamic simulation analysis (MDSA). G6PD variants G410D and V291M which represent classes I and II respectively originating from Asia were chosen for this study and subjected to MDSA. G6PD deficiency clinically manifests into acute hemolytic anemia, chronic non-spherocytic hemolytic anemia (CNSHA), neonatal jaundice and favism, of which variants G410D and V291M are known to exhibit CNSHA and increased microparticle level respectively, indicative of oxidative damage [16,17]. Moreover, these variants have been biochemically characterized, providing an opportunity to bridge our *in silico* analyses with reports from previous *in vitro* experiments that examine G6PD protein-ligand affinity [17,18].

The human G6PD enzyme, in its active state is found in dynamic equilibrium of dimer and tetramer [19,20]. However, there are no complete monomeric or dimeric structures of the human G6PD protein bound to substrate glucose-6-phosphate (G6P) with the structural NADP (s.NADP) and catalytic cofactor NADP (c.NADP) available in the Protein Data Bank [21]. In order to study the structural changes of G6PD mutants, a complete G6PD monomer in complex with its ligands was constructed using the AutoDock 4.2 program [22]. Crystal structures 2BH9 and 2BHL were retrieved from the Protein Data Bank [20]. A complete monomer was produced by docking G6P from 2BHL onto 2BH9, thereby producing a complete G6PD monomer. Site-directed mutagenesis was performed using the SwissPDB viewer to create the variants [23]. Structural analyses on the protein were performed by using PyMOL [24].

Previous studies have exemplified the power of MDSA in estimating protein-ligand affinities and evaluating protein structural integrity [25–27]. Moreover, there have been G6PD-related MDSA studies which focused on the protein-ligand affinities and structural integrity for G6PD monomers and dimers respectively [28,29]. However, such studies analyzed G6PD variants common to the Arab, USA and German population, there is lack of information for variants common to the Asian population.

The structural stability of the constructed monomeric native protein and variants were analyzed by using the molecular dynamics simulation program GROMACS 4.6.7 with the GROMOS96 54a7 force field [30,31]. The protein structure was solvated in a cubic box and neutralized by using the GROMACS genion tool. The system was neutralized by adding sodium ions. The system’s total energy was minimized until the lowest energy (1000 kJ) was obtained. The system was subsequently equilibrated by subjecting the system to 50,000 steps of NVT and NPT. The system was then subjected to a 100 ns simulation.

The resultant trajectories were analyzed using utilities available in the GROMACS package such as gmx rmsd, gmx rmsf, gmx gyrate, that were used to determine the root-mean-square deviation (RMSD), root mean square fluctuation (RMSF) and radius of gyration (Rg) respectively. The results of the analyses were graphically represented using the XMGRACE software to compare the variants against the native protein or wild type [32].

## 2. Main results

**Fig. 1:**
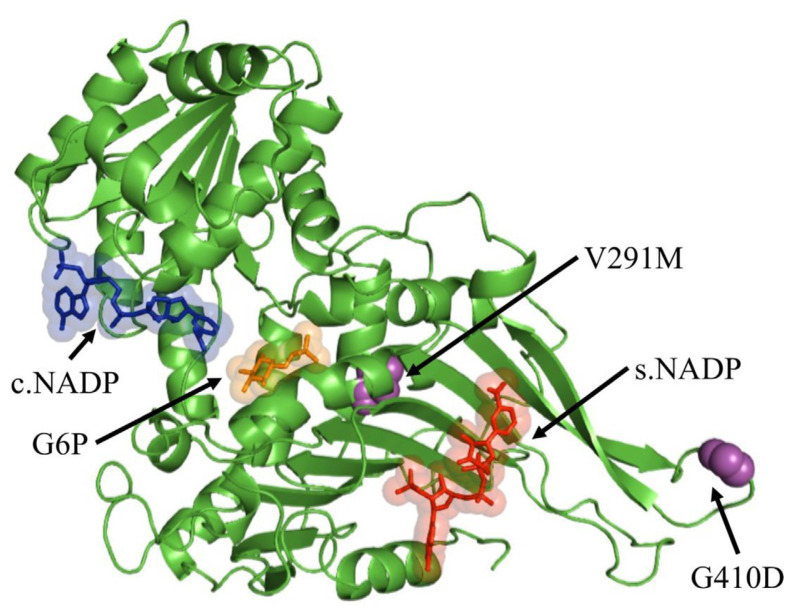
Cartoon view of the G6PD monomer in complex with its substrate glucose-6 phosphate (orange), catalytic NADP (blue), and structural NADP (red). Variants V291M and G410D are depicted in magenta spheres.

Class I Shinagawa (G410D) and class II Viangchan (V291M) had different implications on the enzyme. Results from structural analyses showed G410D was positioned within one of the most flexible coils at the dimer interface close to the structural NADP binding site as depicted in Fig. 1. The increased size of the charged side chain (Asp) resulted in steric hindrance with nearby residues that altered the coil’s structure when glycine was replaced. Lys 407 which is located close to the mutational site is involved in salt bridge formation during dimerization, which is crucial for the enzyme’s catalytic activity [19]. The inactivation of the enzyme’s activity was a result of distorted intermolecular interactions found in G410D. Although the V291M variant is located far from the substrate and structural NADP binding site (22 Ӑ and 25 Ӑ respectively), it causes conformation instability and loss of catalytic efficiency as reported by Boonyuen et al. (2017) [33].

### 2.1. RMSD for wild type G6PD and mutants

**Fig. 2:**
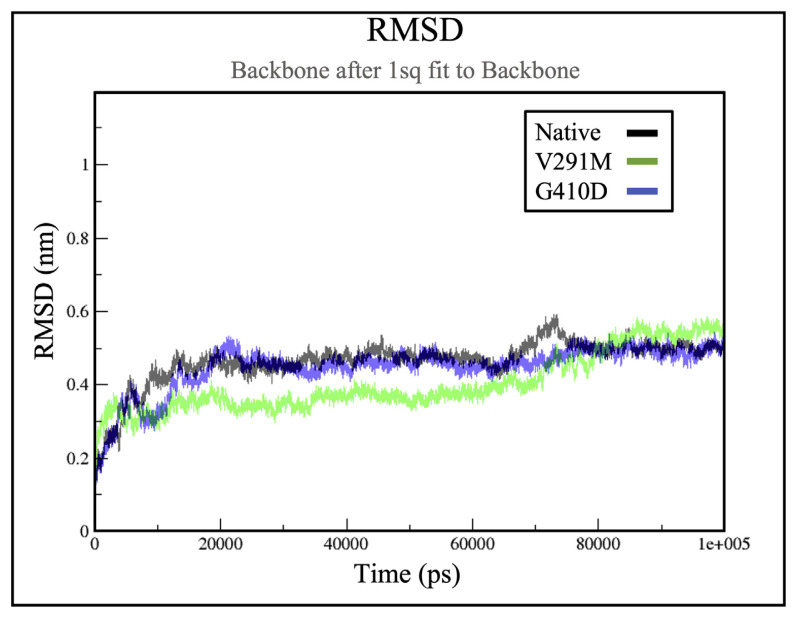
RMSD of protein backbone throughout the simulation. The RMSD of the variants are plotted against the WT. Variants G410D (blue) and V291M (green) are plotted against the native protein (grey).

RMSD allows determining the equilibration of the simulated trajectory throughout the simulation, where a high RMSD indicates greater deviations throughout the simulation [25,34]. From Fig. 2, the RMSD plots indicates that G410D exhibited lower deviations compared to the native protein as the RMSD curve could be stabilized around 0.43 nm throughout the simulation. The native protein and V291M showed significant deviation with average RMSD of 0.45 nm and 0.32 nm respectively. However, the RMSD of V291M increased after 70 ns, and stabilized around 0.5 nm at 90 ns making the native protein structure more stable.

### 2.2. RMSF for wild type G6PD and mutants

**Fig. 3:**
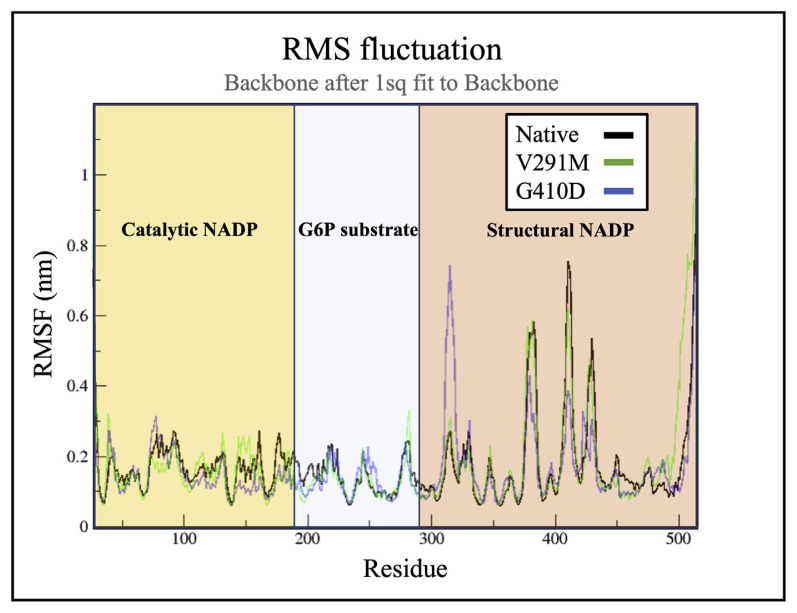
RMSF of the carbon α atoms throughout the simulation for variants G410D (blue) and V291M (green) against the native protein (grey), where the yellow, blue and red layout represent residues which are involved in making the binding pockets of the catalytic NADP, glucose-6-phosphate and structural NADP respectively.

RMSF allows evaluating the differences in flexibility among residues, where higher the RMSF, greater the movements of residues, in relation to their average position [25,34]. The RMSF of G410D fluctuated the most at the dimer interface and the structural NADP binding site. V291M showed high residual fluctuation at the catalytic NADP binding site and at its mutation site compared to the native protein as shown in Fig. 3.

### 2.3. Rg for wild type G6PD and mutants

**Fig. 4:**
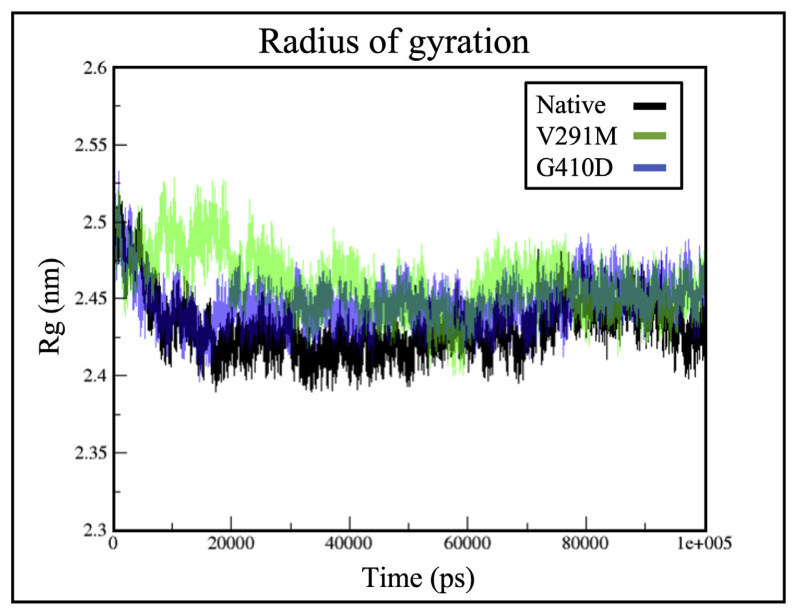
Rg of the protein structure for variants G410D (blue) and V291M (green) against the native protein (black).

Radius of gyration acts as a means to deduce the compactness of a protein structure during simulation, where a low Rg value defines high structural compactness [25,34]. Mutations on the G6PD protein often affect protein folding [35]. As seen in Fig. 4, the gyration plot of G410D showed slight increase in Rg value compared to the native protein indicating that its mutation affected protein folding. V291M on the other hand had higher Rg values compared to the native protein indicating that protein folding was also affected due to its mutation (see Fig. 4).

### 2.4. Hydrogen plots for substrates and cofactors of the wild type G6PD and mutants

**Fig. 5:**
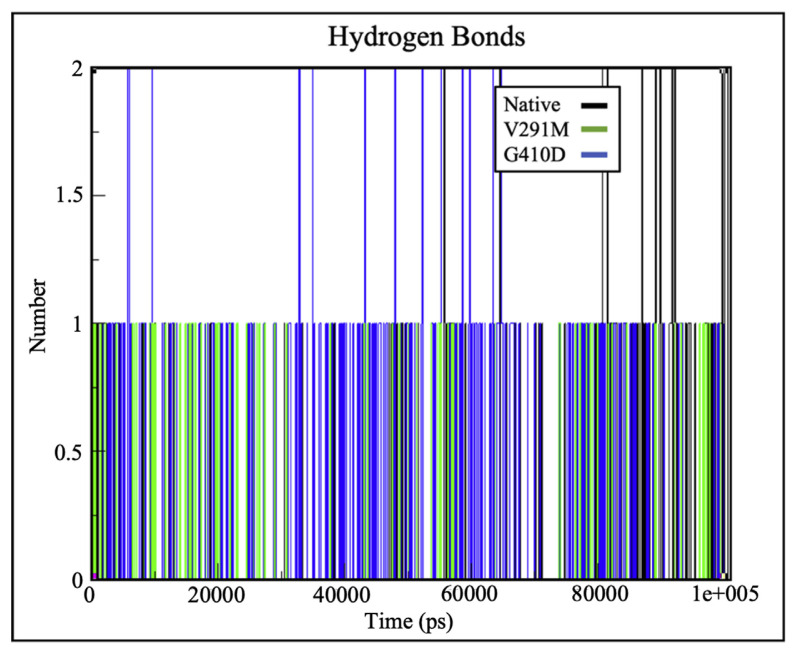
H-bond plots depicting number of hydrogen bonds between G6PD and c.NADP for variants G410D (blue) and V291M (green) against the native protein (black), depicting no significant changes in intermolecular hydrogen binding.

**Fig. 6:**
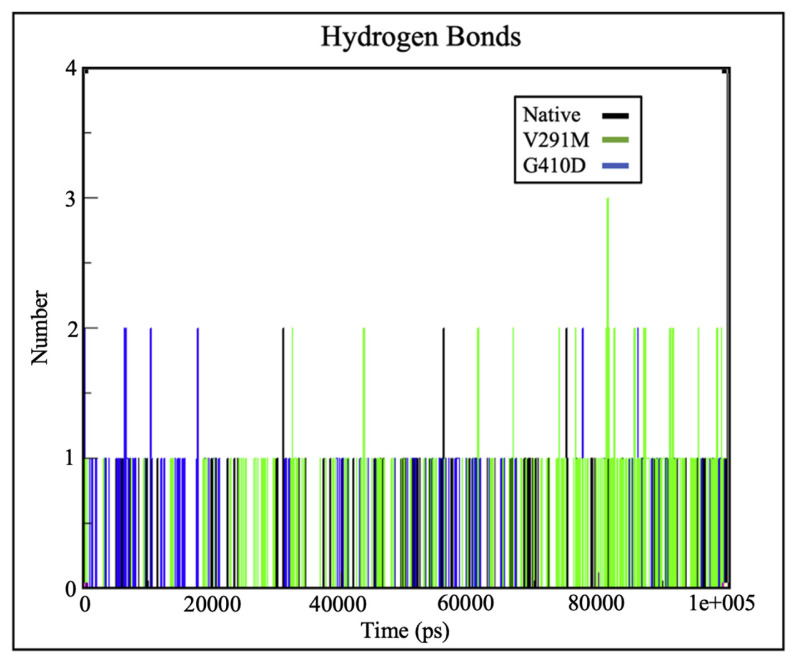
H-bond plots depicting number of hydrogen bonds between G6PD and s.NADP for variants G410D (blue) and V291M (green) against the native protein (black), depicting a change in the number of hydrogen bonds for V291M.

**Fig. 7:**
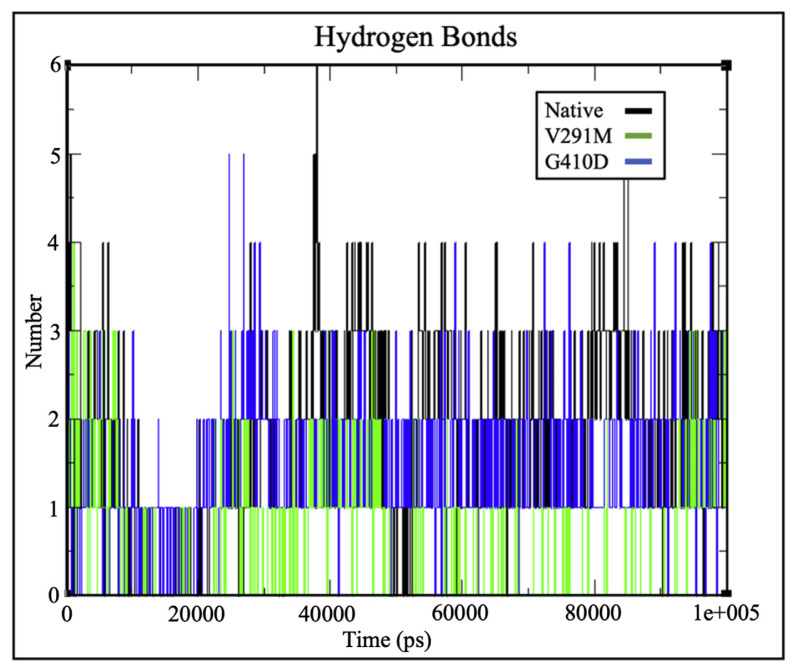
H-bond plots depicting number of hydrogen bonds between G6PD and G6P for variants G410D (blue) and V291M (green) against the native protein (black), depicting fluctuations in the number of hydrogen bonds for both mutants.

Hydrogen bonds play a vital role in molecular recognition and overall stability of protein structures [25,28,34]. Intermolecular hydrogen bonding at the substrate and cofactors were analysed during simulation. From Fig. 5, it can be observed that there were no significant changes in intermolecular hydrogen bonding between the protein and c.NADP for the native protein and variants. From the hydrogen bond plots depicted in Figs. 5 and 6, both native and mutant G6PD structures had similar number of hydrogen bonds to the catalytic and structural NADP ligands respectively. However, the number of hydrogen bonds for G6P differed significantly as shown in Fig. 7, where G410D and V291M appeared to maintain 2 and 1 hydrogen bonds respectively. This indicates that structural changes associated with the mutations might have altered the G6P affinity, hence affecting the enzyme’s catalytic activity induced by hindered substrate oxidation.

Previous kinetic characterization and estimation of protein – ligand affinities for G6PD variants were performed by computing the K_m_ values, which are an inverse measurement of the protein-ligand affinity. Greater the K_m_ values, lower the protein-ligand affinity [36]. Based on the kinetic characterisation by Hirono A et al. (1994) and Gómez-Manzo S A et al. (2016), both G410D and V291M exhibit loss of affinity towards G6P (indicated by higher K_m_ values) which is in accordance with the protein – ligand affinity estimation of this study as shown in Fig. 7. It was interesting to note that there were no significant changes in binding affinity between G6PD and c.NADP for the native protein and variants as shown in Fig. 5, which is similar to the kinetic characteristics for the variants, where they depict similar K_m_ values to the native protein indicating similar c.NADP affinity [17,18].

Invaluable insights on the altered structural stability of the protein was determined from the various analyses performed. Since the Shinagawa variant showed large fluctuations at the dimer interface and the s.NADP binding site, its mutational effect could be translated to understand the structural-functional effects of class I mutants. Moreover, this corroborated previous findings that any mutation affecting the dimerization mechanics of the protein, results in its impaired enzymatic activity, therefore yielding low levels of G6PD which is a major hallmark of class I mutants. Furthermore, the gyration plot displayed a slight increase in Rg value compared to the native structure indicating that the Shinagawa mutation affects protein folding. The Viangchan variant exhibited impaired folding as well, which was characterised by the increase in Rg values. In addition, the RMSF plots depicted low residual fluctuations at the catalytic NADP binding site, whereas a high residual fluctuation was identified at the mutational site. Hence, from the above findings, loss of catalytic activity for the Viangchan variant might have been due to a structural alteration at its mutation site, rather than the dimer interface or the structural NADP binding site.

The effects of the mutations analysed would be more apparent by simulating the G6PD enzyme in its dimeric form. There have been recent discoveries of small molecules which serve as agonists by elevating G6PD enzyme activity in variants [37–40]. One such molecule called AG1 functioned by spanning both monomeric subunits of the G6PD dimer and elevating low enzyme levels in G6PD variants by increasing the structural integrity of the dimer bridge. However, its mode of action appeared to be selective, as it was unable to activate few class I variants [41]. Therefore, constructing and simulating a G6PD dimer in complex with G6P, c.NADP and s.NADP would allow determining whether a particular mutation affects dimerization characterized by increased distance and lack of hydrogen bonds between βN 415–423 of each monomeric subunit.

## 3. Conclusion

Employing computational methods to study how different G6PD variants manifest into different clinical phenotypes allows a better understanding of the structure–function relationship and provides an opportunity to improve diagnostics for G6PD-related diseases and development of drugs for G6PD deficient patients. Future studies on simulating the dimeric form of G6PD would provide more insights on the structural changes of G6PD variants. Following up with *in-vitro* experimentation for variants that show distinct structural activity like protein expression, enzyme kinetic studies and enzyme activity assays to verify computational predictions, would complement this study and make overall findings robust.
